# Effect of the Onset of Intramammary Infection on the Electrical Conductivity of Ewe’s Milk and Study of Various Algorithms for Its On-Line Detection

**DOI:** 10.3390/ani13111808

**Published:** 2023-05-30

**Authors:** Amparo Roca, Raquel Muelas, Manuel Alejandro, Gema Romero, José Ramón Díaz

**Affiliations:** 1 Servicio de Apoyo Técnico a la Docencia y la Investigación, Universidad Miguel Hernández de Elche, 03312 Orihuela, Spain; aroca@umh.es (A.R.); raquel.muelas@umh.es (R.M.); 2 C/Anabel Segura, 7, 28108 Madrid, Spain; manuel_alejandromartinez@yahoo.com; 3 Centro de Investigación e Innovación Agroalimentaria y Agroambiental (CIAGRO), Universidad Miguel Hernández de Elche, 03312 Orihuela, Spain

**Keywords:** mastitis, sensitivity, specificity, milk quality, staphylococcus, streptococcus

## Abstract

**Simple Summary:**

This article studies the effect of the onset of intramammary infection (IMI) in dairy sheep on milk production, electrical conductivity (EC), SCC and milk quality, together with other physiological variation factors. In addition, it analyses the performance of different algorithms that use the variable EC of gland milk (a variable that can be automated in the milking parlour) for economic and early detection of IMI. The onset of IMI was found to cause an increase in SCC and a significant drop in production, more pronounced in bilaterally than unilaterally infected ewes, while EC was significantly higher when infection occurred bilaterally. The best algorithm for IMI detection using EC achieved 50% sensitivity and 100% specificity.

**Abstract:**

The aim of this study was to determine the effect of the onset of intramammary infection (IMI) on the electrical conductivity (EC) of ewe milk and assess the detection capability of various algorithms based on daily glandular milk EC measurement. An experiment was carried out with 26 Manchega sheep located at the farm of the Miguel Hernández University, Elche, Spain. The variables in milk from the gland (production, EC) were monitored daily for 2 weeks during the morning and evening milking; once infection was established in the gland, the variables were measured for a further 4 weeks. In addition, the SCC, sodium, potassium, chloride and milk macro-compositions were analysed. The sensitivity, specificity and positive and negative predictive values for IMI detection of different algorithms were calculated using the EC variable. It was observed that the onset of IMI resulted in an increase in SCC and a significant decrease in yield, and EC rose significantly when infection occurred bilaterally. The best results for IMI detection were obtained with the algorithm that detected deviations greater than 3σ of the conductivity ratio between collateral glands with respect to a moving average calculated with a time horizon of 10 days (50% sensitivity and 100% specificity).

## 1. Introduction

Mastitis in dairy sheep, both clinical and subclinical, causes economic losses due to a drop in production and cheese yield [1]. In a sheep flock with 75% infected glands, a decrease in production of 12.2% was observed [1], whereas in goats, with the same percentage of infected glands, the losses were 2.3%. A production loss related to the increase in SCC was also reported [2], reaching 16% when the threshold of 500 × 103 cells/mL was exceeded, emphasising the importance of implementing SCC control programmes in sheep. Mastitis also causes an alteration in milk composition and a consequent decrease in cheese yield, more pronounced in sheep than in cattle or goats [3]. Another problem associated with subclinical mastitis is that, because it is not detected, no treatment is applied to infected animals, so the disease often persists in subsequent lactations [4]. For this reason, it is of great importance to develop techniques for the early and efficient detection of cases of mastitis in sheep, in order to minimise the economic losses caused by this disease.

The main advantage of measuring EC as a method for detecting mastitis is the ease with which it can be automated by installing EC sensors in short milk tubes. This allows us to record and analyse data from each gland as the ewes are being milked. However, in sheep, there are several non-infectious factors that affect EC (stage of lactation, number of lactations, etc.) in such a way that setting absolute EC thresholds for mastitis detection does not achieve desirable sensitivity or specificity results [5], as is also seen in goats [6] and cows [7]. Accordingly, the collection of multiple EC measurements in the milking parlour is more appropriate than the use of portable equipment and spot measurements to detect high SCC in sheep [8].

To improve the detection of mastitis using the measurement of EC in milk, calculation algorithms capable of obtaining a specificity (Sp) of 95% and sensitivity (Se) of 96 and 100% for clinical and subclinical mastitis, respectively, were developed for cattle, with a confidence interval of 95%, using the tracking signal approach (Tracking Signal Method) [9]. Among the literature consulted, the most commonly used algorithm is based on the comparison of the EC value of the current milking with the moving average of the last milking, setting an increment from which the alarm signal is given. With this method, using the data from the last 14 milkings, Se of 87 and 89% for clinical and subclinical mastitis, respectively, and a Sp of 88% have been obtained [10].

In small ruminants, albeit to a lesser extent, the use of algorithms that include EC for mastitis detection has also been studied. In goats, during the days immediately following the onset of intramammary infection, there was a significant rise in EC which varied in magnitude depending on the causative pathogen [11]. In a subsequent work in goats [12], the use of algorithms that compared the daily and individual measurement of glandular milk EC with its moving average was studied; the authors were able to classify all cases of clinical mastitis, although they obtained different results in subclinical cases depending on the algorithm. The highest Se (58.3%) was obtained by considering those cases in which the EC of the day in question deviated by at least three times the standard deviation of its moving average (calculated with the values of the four previous days) as positive, with 75% Sp. A study, in which EC sensors in commercial liners which allowed for the measurement of EC per gland on-line were used [13], managed to obtain a Se of 80% but Sp between 25 and 42.9%, depending on the calculation algorithm used for mastitis detection in Saanen goats. The same group, in a previous work using the Fuzzy Logic model, had obtained better results: Se of 81% with Sp of 69% [14]. Another study on goats [12] concluded that the best algorithm to choose would depend on the level of prevalence and type of mastitis. In cases of low prevalence and subclinical mastitis (as is common in small ruminants on technified farms), priority should be given to avoiding unnecessary treatment, which implies prioritising the Sp.

In sheep, studies are very scarce. One observed that mastitis causes an increase in milk EC and proposed two thresholds for mastitis detection [15]: one of 5 mS/cm to diagnose glands with mastitis, achieving a Se of 60.2% and a Sp of 91.4%, with 87.9% of the samples correctly classified; the other consists of using a difference of 0.3 mS/cm in EC between the two glands of the same animal, finding better results (70% Se, 93% Sp and 89.1% of samples classified correctly). Another study [16] found no significant differences between the impedance (inverse property of EC) of healthy and infected glands, although they did obtain a negative correlation, but of low value, between impedance and SCC (r = −0.27). In more recent studies, based on the EC measured in samples taken at gland level in Sardinian ewes and using ROC (Receiver Operation Characteristic) analysis, an EC of 4.835 mS/cm was chosen as the discrimination threshold, with which the values of Se, Sp and area under the curve were 73.08%, 75.46% and 0.804%, respectively [17]. They then tested a prototype developed for real-time measurement of EC, with which, using this threshold, they were able to correctly classify 64.7% of the samples that presented SCC > 700,000 cells/mL and 76.5% of samples with low SCC (<700,000 cells/mL), with the authors highlighting the low percentage of false negatives obtained (8.8%).

In order to determine the effect of the onset of intramammary infection on the EC of ewe’s milk, this trial was designed with two objectives in mind: to study the effect of the onset of intramammary infection on the EC of glandular milk and its evolution post-infection and to assess the mastitis detection capability of various algorithms based on daily glandular milk EC measurement.

## 2. Materials and Methods

### 2.1. Experimental Location and Animals Used

The experiment was carried out at the Small Ruminants Training and Experimental Farm of the Higher Polytechnic School of Orihuela, belonging to the Miguel Hernández University (UMH) of Spain. The farming system was intensive, with permanent stabling and a Unifeed diet (consistent throughout the experiment). The reproductive rhythm was one lambing per year, with mating in October–November and lambing scheduled for mid-April. Twenty-six multiparous Manchega ewes were used: 11 ewes at first lambing, 5 ewes at second, 4 ewes at fourth and 6 ewes at fifth parturition. Average prolificacy was 1.54 (±0.51). The ewes suckled the lambs until they were weaned, and the milk not consumed by the lambs was milked once a day to avoid a drop in production. The lambs were weaned at one month of age and the ewes were milked twice a day (8:00 a.m. and 4:00 p.m.) in a Casse 1 × 12 × 12 low-line milking parlour, with the following milking parameters: 36 kPa vacuum level, 180 pulses per minute and 50% pulsation ratio. The glands were milked separately, collecting the milk in volumetric meters.

Feeding of the ewes, consisting of 2.5 kg of special complete mix (Unifeed) for high milk production ewes per ewe per day and administered in two doses, was kept constant throughout the experiment. The animals were also provided with vitamin–mineral blocks as well as cereal straw ad libitum. Water was supplied freely in cup troughs with a constant-level buoy.

### 2.2. Experimental Design and Variables Analysed

The experiment lasted 7 weeks: a 1-week pre-experiment period, used to select the animals, and a 6-week experimental period.

After weaning the lambs, the pre-experimental period began, during which the 50 lactating ewes on the farm were sampled twice at seven-day intervals. In these controls, 5 mL of milk was taken aseptically from each gland before the morning milking and used for bacteriological analysis. Subsequently, the glands of each ewe were milked separately, and the milk was collected in volumetric meters, from which a representative sample of 100 mL was extracted after recording the production. This sample was used to analyse the SCC, EC and macroscopic composition (fat, caseins, whey proteins, lactose and ash) and mineral content (Na+, K+, Cl−). With the information gathered during this period, 26 animals (15 ewes producing less than 400 mL/gland and 11 producing more than 400 mL/gland) free of intramammary infection were selected and used in the experimental period.

The experimental period began three days after the second pre-experimental sampling and lasted six weeks, split into two phases. In the first phase (two weeks), in order to establish conditions prior to the onset of infection, the production and EC of both glands of each animal were measured daily at both milkings (morning and evening). After this initial phase, the animals were subjected to a number of unhealthy situations (UHS) that can occur on commercial farms and increase the likelihood of pathogen entry through the teats during milking. These unfavourable situations were:

(a) Consecutively milking a healthy ewe after another ewe with an intramammary infection, having decreased the diameter of the short milk tubes (thus decreasing the evacuation volume) and plugging liner openings, which favours immersion of the teat in milk and the consequent transmission of infection, while at the same time causing cross-fluctuations by allowing sudden air ingress through a teatcup;
(b) Raising the vacuum level (VL) at milking to 40 kPa;
(c) Overmilking for 3 min;
(d) And, finally, the elimination of teat sealing with iodine after milking.

The glands were subjected to UHS until a sufficient number of cases of infected glands were obtained (Table 1). After resolution of the UHS, the experiment was extended for another four weeks to observe the evolution of the variables studied after the onset of intramammary infection.

Health status definition. The glands were classified according to their health status, based on the results of the bacteriological analysis and SCC. For the classification between positive and negative cultures, the National Mastitis Council recommendations were applied (Harmon et al., 1990). In the positive samples, the microorganisms were isolated and frozen in milk with 2% glycerol at −4 °C until the end of the experiment, at which time, identification of the bacterial genus was performed. Identification was performed using Gram staining and catalase test in colonies with morphology compatible with Gram-positive microorganisms. Finally, in the case of staphylococci, the bacterial species was identified using the Apistah kit (Bio Merieux, France), while those compatible with enterobacteria were identified using the BBL Enterotube II kit (BD Diagnostic Systems, Germany). Glands with positive bacteriological analysis and SCC higher than 400 × 103 cells/mL were classified as infected glands. Glands in which the bacteriological analysis was negative and the SCC was less than 400 × 103 cells/mL were classified as healthy glands. There were no cases in which the bacteriological analysis was positive without SCC elevation or SCC elevations with negative bacteriological analysis.

Bacteriological analysis was performed at different frequencies, depending on the phase of the experiment. Three bacteriological culture studies were performed 14, 7 and 3 days before glands were subjected to UHS and on the same day as the onset of UHS before morning milking. Subsequently, cultures were taken daily to confirm the health status of the glands and the day the infection set in. Once the onset of the infection was confirmed, the experiment was prolonged for 4 more weeks, and 6 cultures were taken at 4, 7, 11, 14, 14, 21 and 28 days after onset of the infection to confirm its presence and/or persistence.

Milk production was determined daily in the morning and evening milkings using volumetric meters, from which a representative sample of the total amount milked (100 mL) was obtained and used to determine the EC and other variables at the frequency set for each milking.

The SCC (×10^3^ cells/mL) was analysed at the Valencian Community Interprofessional Milk Laboratory (Laboratorio Interprofesional de Leche de la Comunidad Valenciana, LICOVAL) using the fluoro-opto-electronic method (Fossomatic 5000; Foss, Hillerød, Denmark) in samples to which azidiol had been added. SCC was determined in milk from the two milkings 14 and 7 days prior to subjecting the glands to UHS, daily from 3 days before to 14 days after the UHS, and four more controls were performed at 18, 21 and 28 days after onset of the infection.

The gross composition (fat, caseins, whey proteins, lactose and ash) and mineral content (Na+, K+, Cl−) were analysed in milk samples taken at the two milkings on days 14, 7 and 3 days before UHS, on the day of the UHS initiation and 4, 7, 11, 14, 18, 21 and 28 days after the onset of infection. The gross composition was analysed using infrared spectroscopy (Milko Scan FT 120; Foss-electric, Hillerød, Denmark), expressing the results as a percentage of wet matter. Na+ and K+ contents (mg/L) were determined using flame photometry (PFP, JENWAY, Staffordshire, UK). To determine the Cl− content (mg/L), the Mohr method adapted for milk (AOAC, 1995) was used, with the analyses always being carried out by the same person to avoid operator effect. Samples were preserved by freezing (−18 °C) until mineral analysis.

EC (mS/cm) was analysed daily at the morning and evening milkings using a laboratory conductivity meter (GLP 32, Crison, Alella, Spain) with automatic temperature compensation at 25 °C.

### 2.3. Statistical Analyses

Effect of the onset of intramammary infection on the variables. To understand the evolution of EC, SCC and gland production as a function of gland health status (INF) over time periods close to the onset of infection (PER), the EC and SCC variables were transformed to logarithm base 10 (LCE, LSCC) to normalise the data distribution. The relationship between LCE and LSCC was studied using a Linear Mixed Model (Proc Mixed, SAS V. 9.2, SAS Institute, Cary, NC, USA, 2012) with the following fixed effects: health status of the glands (INFi, with four levels. 0: Both collateral glands of animals remained free from infection throughout the experiment. 1: Healthy gland during the experiment whose collateral became infected during the experiment. 2: Infected gland whose collateral remained free of infection throughout the experiment. 3: Both collateral glands of bilaterally infected animals during the experiment); the milking session (ORDj, with two levels: morning or evening milking); the time period with respect to the time of onset of infection (PERk, at seven levels; k = 1: from 9 to 7 days before infection; k = 2: from 6 to 4 days before infection; k = 3: from 3 days to 1 day before infection; k = 4: from 1 to 3 days after infection; k = 5: from 4 to 6 days after infection; k = 6: from 7 to 9 days after infection; k = 7: from 10 to 12 days after infection). Finally, the interaction of gland health status with period and milking session was included. As a random effect, the gland (RL: right or left) nested within the ewe (EW) was considered to model the covariance between the observations of the glands within each ewe [18]. A “compound symmetry” type correlation adjustment model of the variance between repeated measures from the same animal was used, given that it was the one that provided the best fit (lowest) according to the Akaike Iteration Criterion (AIC) and the Bayesian Iteration Criterion (BIC).

To analyse the evolution of the variables studied in ewes free of intramammary infection, each healthy animal was assigned a reference infected animal of similar productive level to the one it had before infection and, therefore, the same day of infection (in this case, of non-infection).

Relationship of milk composition and mineral content to EC. To study the relationship between the physicochemical composition of milk and EC, a linear regression was performed using the Proc Reg program of the SAS statistical package (SAS V.9.2. SAS Institute, Cary, NC, USA, 2012). The independent variables were: fat, casein, lactose, whey proteins, chlorides, sodium and potassium. Whey protein, chloride and sodium contents were not significant, so they were discarded from the final model.

Correlation of EC with SCC in milk. The correlation between EC and SCC was studied globally and by SCC intervals (SCC < 400 × 103 cells/mL and SCC ≥ 400 × 103 cells/mL), for which the Proc Corr program of the SAS statistical package (V.9.2. SAS Institute, Cary, NC, USA, 2012) was used.

Study of different computational algorithms for automatic mastitis detection

The study was carried out with the glandular milk EC measurements and the EC ratio between collateral glands (RELEC) (an indicator of the animal’s udder health status) recorded daily. Based on the health status of the ewes’ glands, the RELEC was calculated as follows:

- The ratio between the EC of the infected gland and the healthy gland in animals with unilateral infection;
- The ratio between the EC of the right gland and the EC of the left gland, in the case of healthy or bilaterally infected animals.
- The algorithms tested were:
- EC deviations from the moving average.

Rule 1 (R1): The onset of infection is expected to cause an increase in the EC, so positive cases were classified as those in which the EC was higher than that of the moving average for the previous 4, 8, 10 or 14 days by 5, 10, 20 or 30%, applying the following formula:

EC/Moving average (EC) (4, 8, 10, 14 days) > 1.05; 1,.10; 1.20 or 1.30.

Rule 2 (R2): In the case of RELEC, the onset of a bilateral intramammary infection may result in an increase (infection in the numerator gland) or a decrease (infection in the denominator gland) in the variable. In order to consider both possibilities, positive cases were classified as those in which the RELEC was 5, 10, 15, 20 or 30% higher or lower than the moving average for the previous days. The formula applied was:

0.70; 0.80; 0.90; 0.95 > RELEC/Moving average (RELEC) (4, 8, 10, 14 days) > 1.05; 1.10; 1.20; 1.30.

- Deviations from standard deviation

Deviations from the normal were interpreted as occurring when the variable (EC or RELEC) deviated from the mean (ECmean or RELECmean) by more than 3 or 4 times the standard deviation (σ) of the mean.

Rule 3 (R3): EC - ECmean (4, 8, 10, 14 days) > 3 or 4 σ (4, 8, 10, 14 days).

Rule 4 (R4): In this case, in order to consider both increases and decreases in RELEC that could occur, the cases in which the absolute value of the difference between RELEC and the mean RELEC for the previous 4, 8, 10 or 14 days was greater than 3 or 4 times the σ were deemed positive.

|RELEC—RELECmean (4, 8, 10, 14 days)| > 3 or 4 σ (4, 8, 10, 14 days)

The described algorithms were evaluated by observation during the five days after the onset of infection in two ways. First, the five days were monitored separately. Second, the five days following the onset of infection were considered together: For a case to be classified as negative, the threshold should not be exceeded on any of the five days. If there was at least one positive case within five days from the onset of infection after applying the tested algorithm, the gland was classified as positive. This was intended to prevent the EC elevation caused in the first days after the onset of infection from raising the mean and resulting in false negatives in subsequent days, as it is more difficult to exceed that mean.

Following the application of the different algorithms, positive cases were classified as true positives (TP) if they corresponded to an infected gland and otherwise as false positives (FP). Negative cases were classified as true negatives (TN) when they corresponded to healthy glands and false negatives (FN) when they corresponded to infected glands.

Based on the data obtained for the EC and RELEC variables, the capacity for detection of intramammary infection (sensitivity, specificity, positive predictive value and negative predictive value) was calculated using the algorithms described:

- Sensitivity (Se): The ability of the method to detect positive cases, or the likelihood that a positive sample will be classified as such (TP/(TP + FN)).
- Specificity (Sp): The ability of the method to detect negative cases, or the likelihood that a negative sample will be classified as such (TN/(TN + FP)).
- Positive Predictive Value (PPV): Probability that a sample classified as positive is actually infected (TP/(TP+FP)).
- Negative Predictive Value (NPV): Probability that a sample classified as negative is actually healthy (TN/(TN + FN)).

## 3. Results

### 3.1. Incidence and Prevalence of Infection

The experiment was started with 26 ewes whose glands were free of intramammary infection. After being subjected to UHS, twelve ewes were infected, nine bilaterally and three unilaterally (Table 1), making a total of 21 infected glands; so the prevalence after UHS was 42.87% in glands and 46.15% in ewes. Starting from healthy animals, the incidence throughout the experiment coincides with the prevalence after UHS. All of them were subclinical, with no case of clinical mastitis.

Most of the pathogens isolated (71.43%) belong to the family *Enterobacteriaceae* (Table 2). When infection was established unilaterally, an increase in EC was only observed in the gland in which enterobacteria were isolated, although the increase in EC (0.16 and 0.11 mS/cm in the morning and evening milkings, respectively) was not as high as that observed in ewes infected bilaterally by the same pathogen (0.44 and 0.48 mS/cm in the morning and evening milkings, respectively). In cases where infection occurred bilaterally, a high increase in EC was observed when the infection was caused by *Serratia marcescens* (0.81 mS/cm for evening milking), *Pantogea agglomerans* (0.23 mS/cm in the morning milking and 0.64 mS/cm in the evening), enterobacteria (0.44 and 0.48 mS/cm in the morning and evening milkings, respectively), all considered major pathogens, or by *Staphylococcus* spp. (0.25 mS/cm).

### 3.2. Effect of the Onset of Intramammary Infection on the Variables Studied

The time period with respect to the time of infection onset (PER) had a significant effect on the three variables studied, as did the milking session, the latter being the most responsible for the variations observed in EC and production (Table 3). Regarding SCC, the major factor responsible for its variation was PER, followed by milking session (F = 39.68 and 38.85, respectively).

Udder health status only had a significant effect on SCC, but its interaction with PER and milking session did have a significant effect on all three variables. In the case of EC and production, this indicates that although no significant differences were observed between the different infective stages of the glands, their behaviour did vary throughout the experiment and at each of the two milkings.

Overall, the EC measured in the evening milking was lower than that recorded in the morning milking throughout the experiment and in all four gland types (Table 4).

In the glands that remained healthy throughout the experiment, (INF = 0 and INF = 1) only slight oscillations of the EC were observed throughout the experiment, without any significant variation in the EC due to the reference period of onset of the infection, the variation of the EC being similar in the two milkings in the case of the glands with INF = 0, (animals free of infection in both glands). In glands with INF = 1 (glands free of infection from animals infected in one gland) an upward trend in EC was observed in the evening milking, which was not seen in the morning milking, although no significant differences were found in any milking after the onset of infection in the collateral gland.

In infected glands belonging to ewes that were unilaterally infected (INF = 2), although there was a slight increase in EC in both milkings after the onset of infection, no significant differences were observed between the period before and after the onset of infection. In contrast, in the glands of bilaterally infected ewes, there was a significant increase of 0.17 mS/cm in the morning milking and 0.26 mS/cm in the evening milking in the first period after the onset of infection compared to the previous period. This increase was maintained in the second period after the onset of infection, and then decreased significantly 7 days after infection, to values similar to those recorded in the periods prior to infection.

Regarding gland production (Table 5), a fluctuation in production was observed throughout the experiment in the glands free of infection in both healthy and unilaterally infected animals. It is noteworthy that in the period prior to the reference day of the onset of infection, an increase in production was observed in both groups of glands.

Only in the glands of ewes with bilateral infection was a significant and relevant decrease in production observed after the onset of infection compared to the three previous periods. In the second period after the onset of infection, production returned to the levels recorded in the previous periods, but from the seventh day after the onset of infection, there was a significant decrease, maintained until the end of the experiment in both milkings. In the infected glands of unilaterally infected animals, no effect of the onset of infection in the evening milking was observed. At the morning milking, the variable fluctuated in the periods prior to the onset of infection in a similar way to that observed in glands free of infection, and decreased significantly in the first three days after the onset of infection compared to the previous period, before increasing again in the following period, in a pattern similar to the variations observed in the periods prior to infection.

Contrary to what was observed in EC and production, the SCC measured at the evening milking was generally higher than that recorded in the morning milking (Table 6).

In healthy glands, the SCC fluctuated throughout the periods studied. In the healthy glands of unilaterally infected animals, the SCC did not show a clear trend, varying throughout the experiment with no significant differences between consecutive periods, as was the case with production.

In infected glands, both those belonging to unilaterally infected ewes (INF = 2) and ewes that were bilaterally infected (INF = 3), there was a significant and high increase in SCC in the period following the onset of infection. This increase was maintained in the infected glands of bilaterally infected ewes until the end of the experiment, but in the glands of unilaterally infected ewes in the following periods, the SCC decreased to values similar to, but slightly higher than, those obtained in the periods prior to the onset of infection.

### 3.3. Relationship of EC with the Physicochemical Composition of Milk and Correlation of Milk EC with SCC

A high coefficient of determination (R^2^ = 0.83) of the EC with the physicochemical composition of the milk was found (Table 7), with lactose content being the main factor responsible for the variations observed in the EC, followed by milk casein, potassium and fat contents. The only component that showed a positive relationship with EC was potassium, so a decrease in lactose, casein or milk fat was related to an increase in EC. The remaining milk minerals analysed (chlorides and sodium) did not have a significant effect, so they were discarded from the final model, along with whey protein.

All the correlation coefficients obtained between SCC and EC were significant, with those obtained in the evening milking being higher than those from the morning milking, both globally and in the two SCC intervals (Table 8). In the case of SCC > 400,000 cell/mL, correlation coefficients higher than those recorded for SCC < 400,000 cell/mL were obtained; the highest correlation coefficient between EC and SCC occurred in the evening milking when SCC was higher than 400 × 103 cell/mL (r = 0.47).

### 3.4. Performance of Different Computational Algorithms for Mastitis Detection

When the behaviour of the algorithms was studied separately for the five days following the onset of infection, similar results were obtained for both milking sessions. The increase in the discrimination threshold in all cases led to a decrease in Se and an increase in Sp, regardless of the rule applied or the time horizon considered for the calculation of the mean and σ. The Se obtained by applying the rules to the RELEC was higher than that obtained by applying them to the EC, contrary to what was observed in the Sp. At both milking sessions (morning and evening), the best results were achieved when applying the R2 with a threshold of 5% and considering a time horizon of 14 days (Se of 47.7% and 51.1% and Sp of 77.1% and 86.2%, at morning and evening milkings, respectively), but despite this, there would still be around 30% of cases (both positive and negative) incorrectly classified.

The results obtained when considering the five days after the onset of infection together are better than those obtained when considering the five days separately. In general, the same behaviour is observed in both milking sessions (morning and evening) as when considering the data separately: as the alarm threshold increases, there is a decrease in the Se of the methods, which is accompanied by an increase in the Sp. At the morning milking, better results were obtained than at the evening milking (Table 9). In the morning milking, higher Se and Sp were obtained when applying R1 with a threshold of 5%. Regardless of the time horizon, the values recorded are around 70% for Se and Sp, the same as the values for PPV and NPV. On this occasion, R2 obtained higher Se (91.7%) but lower Sp than R1 (45.5–63.6%). The best results were obtained by applying R4 with a discrimination threshold of 3σ and a time horizon of 10 days, achieving Se of 91.7% and Sp of 90.9%, with PPV and NPV also around 90%.

## 4. Discussion

The incidence and prevalence of intramammary infection was very high (46.15% in ewes and 42.87% in glands), compared to the literature consulted, in which prevalence values range from 5% [19] through 10.4% [20] to 39.9% [21] at gland level and 15% [16] and 32% [22] at the individual level. It was also higher than those obtained by the authors of [5], who obtained an infection rate of 18.75% and 9.52% in multiparous and primiparous ewes, respectively, in the first year and 18.33% in the second year. The high incidence of infection in this experiment is due to the experimental design per se. The various UHSs imposed increased the likelihood of the glands acquiring intramammary infection when the teats were exposed to milking and/or handling practices considered “at risk” for cross-transmission of mastitis. All infections were subclinical, probably due to the fact that bacteriological culture studies were performed from the beginning of the UHS and the SCC was analysed daily, so that when the intramammary infection appeared and before clinical symptoms were observed, the infected glands were no longer subjected to UHS. Most bilateral mastitides were caused by major pathogens, with enterobacteria isolated from 77.78% of infected ewes. This is a very high percentage compared to that reported by [23], who only found enterobacteria in 2.07% of the isolates. The high prevalence of environmental microorganisms is explained by the imposition of UHS, as these practices favour infection by microorganisms located on the teat skin, coming from the bedding or other contaminated objects, being similar to a study [24] that isolated *E. coli* in 41.9% of the cases of mastitis found in 380 Comisana ewes and attributed it to a lack of hygiene and exposure of the ewes to a stressful environment and handling.

The significant effect of milking (morning or evening) on the variables was due to the different intervals between milkings, with 8 h elapsing from the morning milking to the evening and 16 h from the evening milking to the milking the following morning. The higher production in the morning milking generates a dilution effect that causes the SCC to be lower [25,26]. This effect can also occur in the different milk components, affecting its composition and leading to variations in the EC, which has also been observed in cattle [27,28] and sheep reporting an increase in fat at the evening milking [29].

In infected glands belonging to unilaterally infected ewes, no significant variation in EC was observed, similar to what was observed in goats by [11], who attributed it to the fact that for the EC to increase, the infection must cause sufficient tissue damage to modify the permeability of salts to the alveolar lumen, one of the main factors for the increase of EC in milk [30]. The results of the regression study on EC with milk composition as explanatory variables indicate that lactose and caseins are mainly responsible for EC variations (R2partial = 0.81). Both components are synthesised by lactocytes, so that damage to them will cause a decrease in these components [31], which have a negative relationship with the EC.

The onset of bilateral infection caused a significant increase in EC, as observed in several studies in cattle [32,33], in goats [11,34] and in sheep [15,35]. At the morning milkings, the EC increased significantly in the first period after the onset of infection (1–3 days), although the maximum value of EC (4.41 mS/cm) was not reached until the second period after the onset of infection (4–7 days). At the evening milkings, the maximum value (4.26 mS/cm) was reached immediately after the onset of infection. These values are lower than those reported by [15], who observed an EC of 4.40 mS/cm in Manchega ewes free from intramammary infection, reaching values between 5.04 mS/cm and 10.28 mS/cm in glands infected by S. epidermidis and S. aureus, respectively. Additionally, in sheep, a higher EC (5.0 ± 0.56 mS/cm) than those measured in this experiment was found [36], and a high variability in the EC was observed, which was attributed to variations in milk fat content. In more recent studies, an overall mean EC in Sarda ewes of 4.73 ± 0.54 mS/cm, with an EC of 5.20 mS/cm when the SCC was higher than 700 × 103 cells/mL (the threshold used for mastitis classification) and of 4.63 mS/cm in ewes with SCC below this threshold, was recorded [17]. In the same work, the authors proposed a threshold of 4.835 mS/cm to differentiate between healthy and infected glands, with which they obtained 73.08 and 75.46% of Se and Sp, respectively. This threshold is higher than the maximum EC value recorded in this trial, which corroborates the need to consider other factors affecting EC in addition to mastitis (breed, lactation status, number of lambings, etc.). Therefore, the same absolute threshold of EC for the detection of mastitis in all dairy sheep would not be valid [5,8].

The significant effect of the onset of infection on bilaterally infected animals suggests that the use of mastitis detection systems based on daily EC readings could detect increases in the variable and, with it, disease in bilaterally affected glands. In contrast, in animals in which the infection was unilaterally established, in the absence of significant changes in the EC, the disease would not be detected.

Regarding production, variation was recorded during the periods prior to the reference day of infection onset in both the glands of ewes that remained free of infection and those that were unilaterally infected, so the decline observed in the period immediately following the onset of infection appears to be a continuation of normal fluctuations that may be affected by other factors, similar to what was observed by [11]. In this work, unilateral infection was not observed to cause a decrease in the production of the infected gland, nor a compensatory increase in the collateral gland, which contradicts what was previously observed in Manchega sheep by [37,38], who reported a production drop in infected glands close to 50% in both Manchega and Lacaune ewes.

In the glands of bilaterally infected ewes, there was a decrease in production in both milkings in the first period after the onset of infection, dropping from the levels recorded in the periods prior to the onset of infection. This decrease was 21.41 and 19.44% in the morning and evening milkings, respectively, which is greater than what was observed in other studies that quantified production losses due to a bilateral infection caused by major pathogens at 10% [23] and 16% when the SCC exceeded 500 × 103 cells/mL [2].

In Manchega sheep, the SCC values in healthy and infected glands are both higher than those reported in infected glands [39]. As the experiment progressed, a significant increase in SCC was observed in infected glands, especially in the first period after the onset of infection, which is in agreement with the literature consulted [16,21,23,26].

The correlation of SCC with EC was significant, although moderate, in both milkings, being higher in the high-count interval (SCC > 400,000 cells/mL), which shows that EC can be an indicator of gland health status, similar to studies in sheep [8], cattle [32,39] and goats [11].

When applying different calculation algorithms for mastitis detection by daily measurement of EC in the five days after the onset of infection treated separately, moderate Se values were obtained. The cases in which a high Se was obtained were accompanied by low Sp, i.e., many false alarms would be occurring, which would result in unnecessary treatment of healthy animals or the need to use a complementary method to the EC sensors to confirm the disease using colour [40] or lactose, EC and production [41] to achieving good results.

When considering the five days after infection as a whole, high values of Se and Sp were recorded in the morning milkings. Applying R4, which employs the collateral gland EC ratio (RELEC) and 3σ threshold, with a 10-day time horizon, both Se and Sp, PPV and NPV were greater than 90%. Using the same rule, considering the five days separately, we obtained a Se of only 22.7%. This low Se is due to the fact that the onset of intramammary infection causes an increase in EC, which also increases the RELEC. If this increase in RELEC occurs on the first or second day after the onset of infection, it causes an increase in the mean calculated by the algorithm, so that the difference is not large enough for the method to give us the alarm signal, giving rise to FNs.

The authors of [12] studied the use of algorithms that compared the daily and individual measurement of glandular milk EC with its moving average in goats and were able to classify all cases of clinical mastitis, although they obtained different results in subclinical cases depending on the algorithm. The highest Se (58.3%) was achieved by considering the cases in which the EC of the day in question deviated by at least three times the standard deviation from its moving average (calculated with the values of the four previous days) as positive. The authors of [13], in a study in which they included EC sensors in commercial liners that allowed them to measure EC per gland on-line, managed to achieve a Se of 80%, but Sp between 25 and 42.9%, depending on the calculation algorithm used for mastitis detection in Saanen goats. The same group, in a previous work using the Fuzzy Logic model, obtained better results: Se of 81% with a Sp of 69% [14].

In sheep, studies are very scarce; [15] observed that mastitis causes an increase in milk EC and proposed two thresholds for mastitis detection. One of them of 5 mS/cm to diagnose glands with mastitis, achieving a Se of 60.2% and a Sp of 91.4%, with 87.9% of the samples correctly classified. The other threshold consisted of using a difference of 0.3 mS/cm in EC between the two glands of the same animal, finding better results (70% Se, 93% Sp and 89.1% of samples classified correctly). The authors of [16] found no significant differences between the impedance (inverse property of EC) of healthy and infected glands, although they did obtain a negative correlation, but of low value, between impedance and SCC (r = −0.27). In a more recent study [17], based on the EC measured in samples taken at gland level in Sardinian ewes and using ROC analysis, the authors chose an EC of 4.835 mS/cm as the discrimination threshold, with which the values of Se, Sp and area under the curve were 73.08%, 75.46% and 0.804%, respectively. They then tested a prototype developed for real-time measurement of EC, with which, using this threshold, they were able to correctly classify 64.7% of the samples that presented SCC > 700,000 cells/mL and 76.5% of samples with low SCC (<700,000 cells/mL), with the authors highlighting the low percentage of false negatives obtained (8.8%).

## 5. Conclusions

The onset of an intramammary infection leads to an increase in the SCC and a significant drop in production, more pronounced in ewes infected bilaterally than unilaterally. In contrast, milk EC was significantly elevated only when infection occurred bilaterally. The daily measurement of glandular EC (by means of equipment inserted in the short milk tubes that allow the recording of the EC of the glandular milk) would be a good tool for mastitis detection using the appropriate algorithms. The best outcomes were achieved with the rules based on the detection of anomalous variations of RELEC, applying a threshold of 3σ and considering a time horizon of 10 days to calculate the moving average and the set of the five days after onset of the infection. Variations were observed in the performance of the different rules applied depending on the milking session. In general, rules based on deviations from the moving average (R1 and R2) performed better in the morning milking, while those based on deviations from the standard deviation (R3 and R4) performed better in the evening milking. These differences should be considered in the development of systems for the automatic detection of IMI during milking using EC in sheep.

## Figures and Tables

**Table 1:** Health status of the glands before and after being subjected to unfavourable situations for mammary gland health (UHS).

	First Part of the Experiment			Second Part of the Experiment after Subjecting Glands to UHS			
	Healthy	Unilateral infection	Bilateral infection	Healthy	Unilateral infection	Bilateral infection	Total infected
Ewes	23 + 3 ^1^	0	0	11 + 3 ^1^	3	9	12
Glands	49	0	0	28	3	18	21

^1^ Sheep that had only one functional gland.

**Table 2:** EC of infected glands in the 3 days before and 3 days after the onset of infection according to the isolated microorganism.

			EC (mS/cm) ± SD	
INFECTION	Nº of Glands	Milking	3 Days before Infection	3 Days after Infection
**UNILATERAL**				
*S. caprae*	1 (4.76%)	Morning	4.03 ± 0.23	4.06 ± 0.08
		Evening	3.88 ± 0.25	3.87 ± 0.11
*Staphylococcus* spp.	1 (4.76%)	Morning	4.22 ± 0.05	4.14 ± 0.11
		Evening	4.02 ± 0.02	4.13 ± 0.14
*Enterobacterale*s	1 (4.76%)	Morning	**4.01 ± 0.07**	**4.17 ± 0.38**
		Evening	**3.93 ± 0.09**	**4.04 ± 0.18**
**BILATERAL**				
*S. xylosus*	2 (9.52%)	Morning	4.62 ± 0.24	4.62 ± 0.12
		Evening	4.32 ± 0.18	4.32 ± 0.11
*Staphylococcus* spp.	2 (9.52%)	Morning	**3.93 ± 0.05**	**4.07 ± 0.22**
		Evening	**3.73 ± 0.09**	**3.98 ± 0.23**
*Serratia marcescens*	2 (9.52%)	Morning	4.87 ± 0.13	4.84 ± 0.81
		Evening	**4.49 ± 0.11**	**5.30 ± 1.76**
*Klebsiella pneumoniae*	4 (19.05%)	Morning	4.54 ± 0.14	4.47 ± 0.28
		Evening	4.22 ± 0.15	4.24 ± 0.19
*Pantogea agglomerans*	2 (9.52%)	Morning	**3.20 ± 0.16**	**3.43 ± 0.23**
		Evening	**3.25 ± 0.16**	**3.89 ± 0.24**
Other *Enterobacterales*	6 (28.57%)	Morning	**4.18 ± 0.28**	**4.62 ± 0.59**
		Evening	**3.97 ± 0.19**	**4.45 ± 0.72**

**Table 3:** Results of the statistical analysis (F-value and significance level) of the variables LCE, LSCC and gland milk production of infected glands in the 3 days before and 3 days after the onset of infection according to the isolated microorganism.

	lEC		LSCC		Production	
EFFECT	F	SL	F	SL	F	SL
Period	6.09	<0.0001	39.68	<0.0001	12.12	<0.0001
Milking	86.30	<0.0001	38.85	<0.0001	593.74	<0.0001
Infection	0.22	0.8840	8.24	<0.0001	1.50	0.2133
Infection × Period × Milking	3.56	<0.0001	14.20	<0.0001	2.61	<0.0001

**Table 4:** Evolution of LEC (mean ± standard error, antilogarithm of LEC (mS/cm)) according to the health status of the glands and the period relative to the onset of infection.

	Morning Milking						
INF	Days Prior to Infection			Days after Infection			
	9-7	6-4	3-1	1-3	4-6	7-9	10-12
0	0.6194 ^ab^	0.6194 ^ab^	0.6227 ^a^	0.6194 ^ab^	0.6194 ^ab^	0.6194 ^ab^	0.6131 ^b^
	±0.0101	±0.0101	±0.0101	±0.0102	±0.0102	±0.0101	±0.0101
	(4.16) *	−4.15	−4.19	−4.16	−4.15	−4.13	−4.1
1	0.6101	0.617	0.6001	0.606	0.5957	0.6108	0.6062
	±0.0229	±0.0231	±0.0237	±0.0231	±0.0231	±0.0229	±0.0229
	−4.07	−4.14	−3.98	−4.04	−3.94	−4.08	−4.04
2	0.6091	0.6074	0.6087	0.6151	0.6145	0.6199	0.6092
	±0.0229	±0.0231	±0.0237	±0.0229	±0.0229	±0.0229	±0.0229
	−4.07	−4.05	−4.06	−4.12	−4.12	−4.17	−4.07
3	0.6111 ^a^	0.6141 ^a^	0.6250 ^b^	0.6413 ^c^	0.6446 ^c^	0.6242 ^b^	0.6307 ^b^
	±0.0125	±0.0125	±0.0125	±0.0125	±0.0125	±0.0125	±0.0125
	−4.08	−4.11	−4.22	−4.38	−4.41	−4.21	−4.27
	Evening milking						
INF	Days prior to infection			Days after infection			
	9-7	6-4	3-1	1-3	4-6	7-9	10-12
0	0.6018 ^a^	0.6064 ^a^	0.6060 ^a^	0.6036 ^a^	0.5955 ^b^	0.6001 ^ab^	0.5955 ^b^
	±0.0102	±0.0101	±0.0102	±0.0101	±0.0102	±0.0101	±0.0101
	(4.00) *	−4.04	−4.04	−4.01	−3.94	−3.98	−3.94
1	0.5673 ^a^	0.5978 ^bcd^	0.6094 ^bd^	0.6119 ^b^	0.6122 ^b^	0.5759 ^ac^	0.5874 ^ad^
	±0.0229	±0.0231	±0.0237	±0.0231	±0.0231	±0.0229	±0.0229
	−3.69	−3.96	−4.07	−4.09	−4.09	−3.77	−3.87
2	0.5872	0.5906	0.5955	0.6032	0.6066	0.5932	0.5946
	±0.0229	±0.0231	±0.0237	±0.0229	±0.0229	±0.0229	±0.0229
	−3.87	−3.9	−3.94	−4.01	−4.04	−3.92	−3.93
3	0.5953 ^a^	0.6041 ^ab^	0.6017 ^ab^	0.6289 ^c^	0.6221 ^c^	0.6099 ^b^	0.6033 ^ab^
	±0.0126	±0.0125	±0.0125	±0.0125	±0.0126	±0.0125	±0.0125
	−3.94	−4.02	−4	−4.26	−4.19	−4.07	−4.01

INF: Glandular health status. 0: Both collateral glands free of infection throughout the experiment. 1: Free of infection throughout the experiment whose collateral was infected during the experiment. 2: Infected during the experiment whose collateral remained free of infection. 3: Both collateral glands infected during the experiment. (*) LEC antilogarithm. (mS/cm). ^abcd^ Different superscripts in the same row indicate significant differences (*p* < 0.05).

**Table 5:** Evolution of production (mL) (mean ± standard error) according to the health status of the glands and the period relative to the onset of infection.

	Morning Milking						
INF	Days Prior to Infection			Days after Infection			
	9-7	6-4	3-1	1-3	4-6	7-9	10-12
0	367.38 ^abc^	343.49 ^acd^	388.66 ^b^	334.10 ^ade^	353.78 ^a^	300.86 ^e^	315.46 ^de^
	±35.90	±35.87	±35.87	±35.89	±35.90	±35.87	±35.87
1	471.11	431.44	452.59	418.92	369.81	415.56	376.44
	±88.79	±89.69	±92.58	±89.69	±89.69	±88.79	±88.79
2	686.67 ^ab^	560.84 ^c^	722.54 ^a^	539.44 ^cd^	636.67 ^ac^	565.00 ^c^	441.67 ^bd^
	±88.79	±89.69	±92.57	±88.79	±88.79	±88.79	±88.79
3	420.09 ^a^	412.04 ^a^	439.44 ^a^	345.35 ^b^	403.52 ^a^	319.44 ^b^	342.15 ^b^
	±43.40	±43.40	±43.40	±43.45	±43.40	±43.40	±43.40
	Evening milking						
INF	Days prior to infection			Days after infection			
	9-7	6-4	3-1	1-3	4-6	7-9	10-12
0	210.74 ^ab^	198.30 ^ab^	215.18 ^a^	190.34 ^ab^	191.81 ^ab^	179.29 ^b^	222.41 ^ab^
	±35.87	±35.87	±35.87	±35.89	±35.87	±35.87	±35.87
1	335.00 ^a^	233.81 ^ab^	287.25 ^ab^	278.30 ^ab^	232.92 ^b^	230.67 ^b^	231.56 ^b^
	±88.79	±89.69	±92.58	±89.69	±89.69	±88.79	±88.79
2	403.33 ^a^	308.34 ^ab^	315.21 ^ab^	328.78 ^ab^	274.44 ^b^	299.33 ^b^	267.78 ^b^
	±88.79	±89.69	±92.57	±88.79	±88.79	±88.79	±88.79
3	257.28 ^a^	251.61 ^ac^	260.81 ^a^	208.71 ^b^	221.88 ^ab^	196.04 ^b^	211.69 ^bc^
	±43.45	±43.40	±43.40	±43.45	±43.40	±43.40	±43.40

INF: Glandular health status. 0: Both collateral glands free of infection throughout the experiment. 1: Free of infection throughout the experiment whose collateral was infected during the experiment. 2: Infected during the experiment whose collateral remained free of infection. 3: Both collateral glands infected during the experiment. ^abcde^ Different superscripts in the same row indicate significant differences (*p* < 0.05).

**Table 6:** Evolution of LSCC (10^3^ cel/mL, mean ± standard error) according to the health status of the glands and the period relative to the onset of infection.

	Morning Milking						
	Days Prior to Infection			Days after Infection			
INF	9-7	6-4	3-1	1-3	4-6	7-9	10-12
0	1.9208 ^ab^	1.8760 ^a^	1.8897 ^a^	2.1006 ^b^	1.9991 ^ab^	2.0127 ^ab^	1.9714 ^ab^
	±0.0126	±0.0856	±0.0749	±0.0752	±0.0754	±0.0749	±0.0752
	(83.33) *	−75.16	−77.57	−126.07	−99.79	−102.97	−93.63
1	1.9844	2.0133	1.928	2.1553	2.3386	2.1215	2.3242
	±0.2699	±0.2438	±0.2156	±0.2156	±0.1909	±0.1909	±0.1909
	−96.47	−103.11	−84.72	−142.99	−218.07	−132.28	−210.96
2	2.2019 ^ac^	2.2950 ^ac^	2.2024 ^a^	3.4904 ^b^	2.5748 ^ac^	2.7265 ^c^	2.5922 ^ac^
	±0.2699	±0.2437	±0.2154	±0.1969	±0.1909	±0.1909	±0.1909
	−159.18	−197.24	−159.37	(3.093.14)	−375.66	−532.72	−391.02
3	1.634 ^a^	1.6885 ^a^	1.6730 ^a^	3.4273 ^b^	2.5172 ^c^	2.4537 ^c^	2.2613 ^d^
	±0.1240	±0.1013	±0.0908	±0.0908	±0.0902	±0.0902	±0.0905
	−42.99	−48.81	−47.1	(2.674.85)	−329	−284.25	−182.52
	Evening milking						
	Days prior to infection			Days after infection			
INF	9-7	6-4	3-1	1-3	4-6	7-9	10-12
0	2.1487 ^abc^	2.0911 ^ac^	2.0562 ^a^	2.2897 ^b^	2.2262 ^bc^	2.1100 ^ac^	2.1285 ^ac^
	±0.1026	±0.0862	±0.0754	±0.0748	±0.0749	±0.0748	±0.0749
	(140.83) *	−123.34	−113.82	−194.85	−168.34	−128.82	−134.43
1	2.3875	2.4155	2.2372	2.3799	2.5368	2.3476	2.5762
	±0.3179	±0.2699	±0.2280	±0.21561	±0.20431	±0.1909	±0.1970
	−244.06	−260.32	−172.66	−239.83	−344.19	−222.64	−376.88
2	2.5034 ^a^	2.6141 ^a^	2.5281 ^a^	3.5394 ^b^	2.6991 ^a^	2.9043 ^a^	2.8518 ^a^
	±0.2699	±0.2699	±0.2154	±0.1909	±0.1909	±0.1909	±0.1969
	−318.71	−411.24	−337.36	−3462.58	−500.15	−802.23	−710.89
3	1.9015 ^a^	1.9022 ^a^	1.9335 ^a^	3.6772 ^b^	2.5636 ^c^	2.6009 ^c^	2.3974 ^d^
	±0.124	±0.1021	±0.0912	±0.0916	±0.0908	±0.09122	±0.09052
	−79.71	−79.84	−85.8	−4755.54	−366.1	−398.93	−249.69

INF: Glandular health status. 0: Both collateral glands free of infection throughout the experiment. 1: Free of infection throughout the experiment whose collateral was infected during the experiment. 2: Infected during the experiment whose collateral remained free of infection. 3: Both collateral glands infected during the experiment. (*) LSCC Antilogarithm (×10^3^ cel/mL). ^abcd^ Different superscripts in the same row indicate significant differences (*p* < 0.05).

**Table 7:** Relationship of EC to milk composition and mineral content.

Variables	Statistic			Model
	Parameter	SE	Partial R^2^	
Intercept	1.7280	±0.0497		N = 1872 R^2^ = 0.83 *p*-value: <0.0001 F-value: 193.63
LACTOSE (%)	−0.1982	±0.0099	0.4325	
CASEIN (%)	−0.0544	±0.0041	0.3871	
POTASSIUM (mg/L)	0.00002	±0.00001	0.0049	
FAT (%)	−0.0032	±0.0015	0.0050	

SE: Standard error; R^2^: Coefficient of determination; N: Number of observations.

**Table 8:** Correlation of EC and SCC.

SCC Interval (×10^3^ cell/mL)	Milking				Total	
	Morning		Evening			
	r	*n*	r	*n*	r	*n*
SCC < 400	0.12 ***	705	0.15 **	663	0.06 *	1368
SCC ≥ 400	0.39 ***	131	0.47 ***	156	0.43 ***	287
Total	0.30 ***	836	0.39 ***	819	0.33 ***	1655

r: Correlation coefficient; *n*: Number of observations used. * *p* < 0.05; ** *p* < 0.01; *** *p* < 0.001.

**Table 9:** Se, Sp, PPV and NPV results when applying the algorithms * to the EC measurements, considering the 5 days post-infection jointly.

Morning Milking													
Time Horizon		R1				R2				R3		R4	
		5%	10%	20%	30%	5%	10%	20%	30%	3σ	4σ	3σ	4σ
4 days	Se (%)	77.3	36.4	18.2	13.6	91.7	58.3	25.0	0.0	40.9	36.4	75.0	58.3
	Sp (%)	70.4	92.6	96.3	96.3	54.5	100.0	100.0	100.0	81.5	85.2	36.4	81.8
	PPV (%)	68.0	80.0	80.0	75.0	68.8	100.0	100.0	0.0	64.3	66.7	56.3	77.8
	NPV (%)	79.2	64.1	59.1	57.8	85.7	68.8	55.0	47.8	62.9	62.2	57.1	64.3
8 days	Se (%)	72.7	45.5	18.2	13.6	91.7	58.3	25.0	0.0	31.8	27.3	91.7	58.3
	Sp (%)	77.8	92.6	96.3	96.3	45.5	100.0	100.0	100.0	92.6	96.3	63.6	90.9
	PPV (%)	72.7	83.3	80.0	75.0	64.7	100.0	100.0	0.0	77.8	85.7	73.3	87.5
	NPV (%)	77.8	67.6	59.1	57.8	83.3	68.8	55.0	47.8	62.5	61.9	87.5	66.7
10 days	Se (%)	72.7	40.9	18.2	13.6	91.7	50.0	25.0	0.0	36.4	22.7	91.7	66.7
	Sp (%)	85.2	92.6	96.3	96.3	63.6	100.0	100.0	100.0	96.3	96.3	90.9	90.9
	PPV (%)	80.0	81.8	80.0	75.0	73.3	100.0	100.0	0.0	88.9	83.3	91.7	88.9
	NPV (%)	79.3	65.8	59.1	57.8	87.5	64.7	55.0	47.8	65.0	60.5	90.9	71.4
14 days	Se (%)	77.3	45.5	18.2	13.6	91.7	58.3	25.0	0.0	31.8	27.3	75.0	58.3
	Sp (%)	74.1	96.3	96.3	96.3	45.5	100.0	100.0	100.0	96.3	96.3	90.9	100.0
	PPV (%)	70.8	90.9	80.0	75.0	64.7	100.0	100.0	0.0	87.5	85.7	90.0	100.0
	NPV (%)	80.0	68.4	59.1	57.8	83.3	68.8	55.0	47.8	63.4	61.9	76.9	68.8
Evening milking													
Time horizon		R1				R2				R3		R4	
		5%	10%	20%	30%	5%	10%	20%	30%	3σ	4σ	3σ	4σ
4 days	Se (%)	72.7	31.8	13.6	13.6	91.7	50.0	16.7	16.7	45.5	40.9	83.3	50.0
	Sp (%)	70.4	96.3	96.3	100	36.4	90.9	100	100	92.6	96.3	45.5	72.7
	PPV (%)	66.7	87.5	75.0	100	61.1	85.7	100	100	83.3	90.0	62.5	66.7
	NPV (%)	76.0	63.4	57.8	58.7	80.0	62.5	52.4	52.4	67.6	66.7	71.4	57.1
8 days	Se (%)	68.2	36.4	13.6	13.6	91.7	50.0	16.7	8.3	45.5	36.4	41.7	25.0
	Sp (%)	85.2	96.3	100	100	36.4	100	100	100	96.3	100	90.9	100.0
	PPV (%)	78.9	88.9	100	100	61.1	100	100	100	90.9	100	83.3	100.0
	NPV (%)	76.7	65.0	58.7	58.7	80.0	64.7	52.4	50.0	68.4	65.9	58.8	55.0
10 days	Se (%)	63.6	40.9	13.6	13.6	91.7	58.3	16.7	8.3	40.9	31.8	50.0	33.3
	Sp (%)	85.2	96.3	100	100	45.5	100	100	100	100	100	100	100
	PPV (%)	77.8	90.0	100	100	64.7	100	100	100	100	100	100	100
	NPV (%)	74.2	66.7	58.7	58.7	83.3	68.8	52.4	50.0	67.5	64.3	64.7	57.9
14 days	Se (%)	68.2	40.9	18.2	13.6	100.0	58.3	16.7	8.3	31.8	18.2	33.3	16.7
	Sp (%)	70.4	92.6	100	100	54.5	100	100	100	100	100	100	100
	PPV (%)	65.2	81.8	100	100	70.6	100	100	100	100	100	100	100
	NPV (%)	73.1	65.8	60.0	58.7	100.0	68.8	52.4	50.0	64.3	60.0	57.9	52.4

* See Section 2 for the definition of the rules. Se: Sensitivity; Sp: Specificity; PPV: Positive Predictive Value; NPV: Negative Predictive Value.

## Data Availability

Not applicable.
